# World Dengue Day: A call for action

**DOI:** 10.1371/journal.pntd.0010586

**Published:** 2022-08-04

**Authors:** Nattachai Srisawat, Usa Thisyakorn, Zulkifli Ismail, Kamran Rafiq, Duane J. Gubler, on behalf of ADVA-ISNTD World Dengue Day Committee

**Affiliations:** 1 Tropical Medicine Cluster, Chulalongkorn University, Excellence Center for Critical Care Nephrology, King Chulalongkorn Memorial Hospital, and Center of Excellence in Critical Care Nephrology, Chulalongkorn University, Bangkok, Thailand; 2 Tropical Medicine Cluster, Chulalongkorn University and Faculty of Tropical Medicine, Mahidol University, Bangkok, Thailand; 3 Department of Pediatrics, KPJ Selangor Specialist Hospital, Selangor, Malaysia; 4 International Society for Neglected Tropical Diseases, London, United Kingdom; 5 Program in Emerging Infectious Diseases, Duke-NUS Medical School, Singapore; National University Singapore Saw Swee Hock School of Public Health, SINGAPORE

## Abstract

Commemorating the 2021 ASEAN Dengue Day and advocacy for World Dengue Day, the International Society for Neglected Tropical Diseases (ISNTD) and Asian Dengue Voice and Action (ADVA) Group jointly hosted the ISNTD-ADVA World Dengue Day Forum–Cross Sector Synergies in June 2021. The forum aimed to achieve international and multisectoral coordination to consolidate global dengue control and prevention efforts, share best practices and resources, and improve global preparedness. The forum featured experts around the world who shared their insight, research experience, and strategies to tackle the growing threat of dengue. Over 2,000 healthcare care professionals, researchers, epidemiologists, and policy makers from 59 countries attended the forum, highlighting the urgency for integrated, multisectoral collaboration between health, environment, education, and policy to continue the march against dengue. Sustained vector control, environmental management, surveillance improved case management, continuous vaccine advocacy and research, capacity building, political commitment, and community engagement are crucial components of dengue control. A coordinated strategy based on science, transparency, timely and credible communication, and understanding of human behavior is needed to overcome vaccine hesitancy, a major health risk further magnified by the COVID-19 pandemic. The forum announced a strong call to action to establish World Dengue Day to improve global awareness, share best practices, and prioritize preparedness in the fight against dengue.

## Author summary

The growing threat of dengue poses a significant public health burden endangering the well-being and socioeconomic development of many countries across the world. The International Society for Neglected Tropical Diseases (ISNTD) and Asian Dengue Voice and Action (ADVA) group brought together the collaborative efforts of healthcare care professionals, researchers, epidemiologists, environmentalists, and policy makers to coordinate international dengue control strategy. A strong call to action to establish a World Dengue Day was announced to improve global awareness, share best practices, and prioritize preparedness in the fight against dengue. The World Dengue Day highlighted the need for all governments, healthcare professionals, civil societies, public and private sectors, schools and universities, and citizens in dengue-endemic countries to form a strong coalition to encourage and accelerate a collective dengue control response.

## Introduction

**Fig 1:**
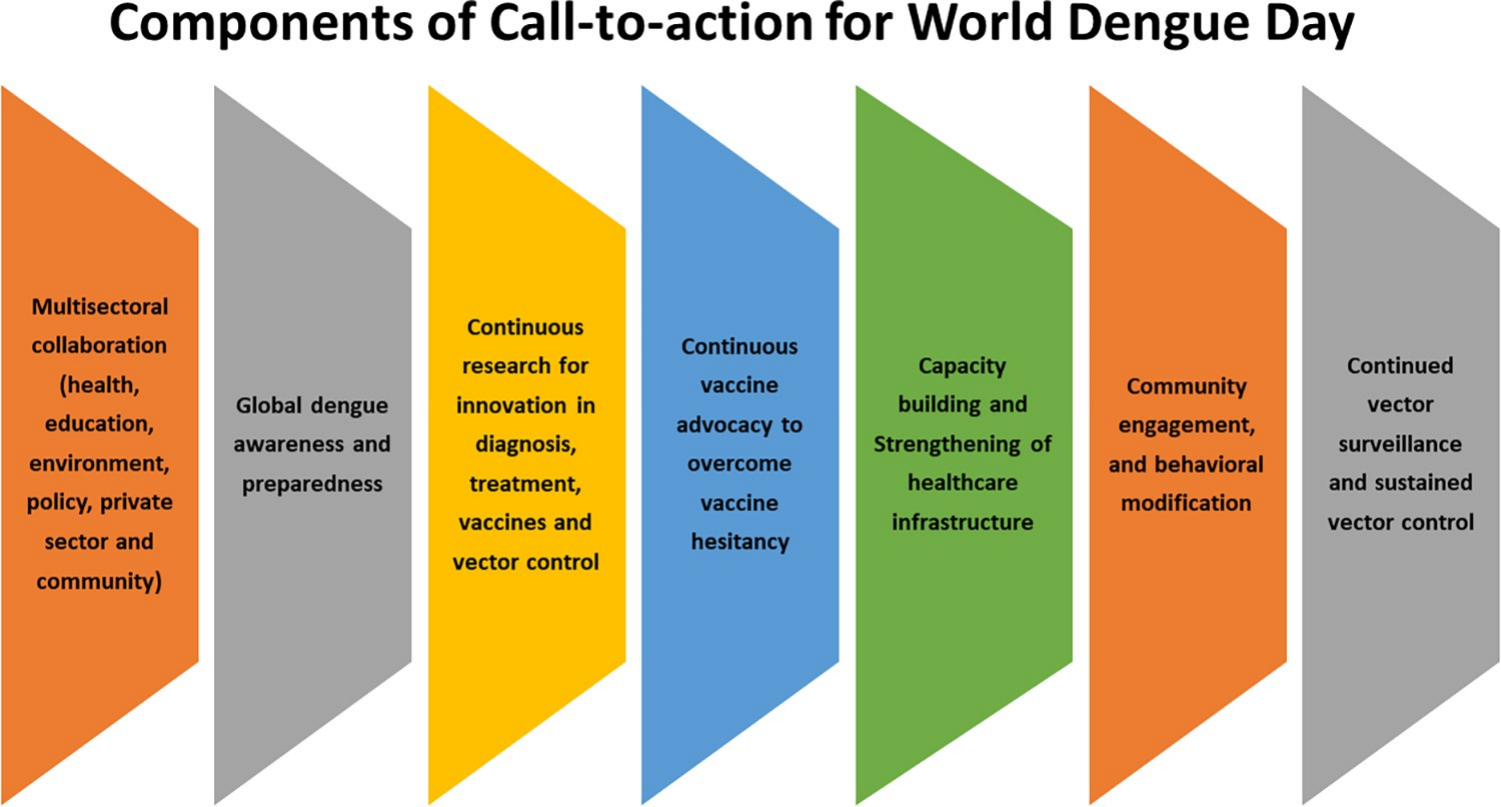
Call to action for World Dengue Day.

Dengue is a global public health burden affecting over 120 countries. An alarming 5.2 million dengue cases were recorded in 2019 [1]. Though 50% of the world’s population is at risk of dengue, Asia contributes 70% of the global dengue burden [1]. The World Health Organization (WHO) target for 2021–2030 Global Strategy for Dengue Prevention and Control is to reduce the dengue case fatality rate to 0% by 2030 [2]. To achieve this target, dengue must be acknowledged as a collective threat. Global collaborative efforts are needed to strengthen dengue preparedness, prevention, and control. Commemorating the 2021 ASEAN Dengue Day and advocacy for World Dengue Day, the International Society for Neglected Tropical Diseases (ISNTD) and Asian Dengue Voice and Action (ADVA) Group jointly hosted the ISNTD-ADVA World Dengue Day Forum–Cross Sector Synergies in June 2021. The international forum highlighted the urgent need for multisectoral partnerships between health, environment, and education ministries, the private sector, and the community for effective integrated dengue control across the globe. The forum called on the United Nations and WHO to establish a World Dengue Day to prioritize global awareness and preparedness in the fight against dengue (Fig 1). The petition for World Dengue Day has to date gathered over 29,000 signatures from dengue stakeholders across 110 countries (https://www.isntd.org/world-dengue-day).

### Diagnostic and therapeutic targets for dengue

Though most dengue patients do not enter the critical stage, there is uncertainty on which patients might progress to severe dengue. Identification of imminent severe dengue is crucial to allow timely management of shock and bleeding, facilitate early referrals, and reduce mortality [3]. Availability of a predictive test for severe dengue that is simple, sensitive, and can provide rapid point of care diagnosis will avoid unnecessary hospitalization, save resources, and reduce time spent on repeated laboratory tests [3]. To inform healthcare decisions for timely clinical management, the biomarker should be applied early upon hospital admission to patients at risk of progressing to severe dengue, especially in dengue warning sign patients. However, such a test is currently unavailable. There are several potential clinical biomarkers that need to be explored further to allow timely recognition of severe dengue. Continued research, funding, and resource allocation are crucial for development of such predictive tests. Using validation cohorts from 8 countries across 3 continents, Robinson and colleagues identified a 20-gene set that can predict progression to severe dengue. Its predictive capacity is independent of virus strain, patient’s age, and genetic background and performs well in immunosuppressed patients and pregnant patients. In the Colombia cohort, the 20-gene set showed 100% sensitivity and 76% to 79% specificity; however, it needs to be validated in large prospective cohorts [4]. In fact, genetic testing is costly. It is a high technology that requires staff with good training and high experience in molecular biology. Implementation of the gene-based test for predictive of severe dengue requires a platform able to quantitatively measure the level of several genes in whole blood samples and ideally incorporate an integral, rapid RNA extraction step [5]. While a few platforms have been developed that enable multiplexing of a large number of genes based on quantitative reverse transcription (qRT)-PCR within only a few hours [6,7], the high manufacturing cost currently challenges their use in resource-limited settings [5].

Secondly, lack of specific treatment for dengue infection highlights the compelling need for innovative therapeutic strategies. Advancing research on the role of inflammatory mediators in dengue pathogenesis can offer potential therapeutic targets. The dengue nonstructural protein 1 (NS1) plays a crucial part in pathogenesis of dengue through induction of endothelial hyperpermeability, RNA replication, and immune evasion and can serve as a potential therapeutic target for severe dengue. NS1 stimulates inflammatory cytokines (e.g., IL-6, IL-10), other mediators (e.g., TNF-α, IL-1β, and IL-8), and noncytokine mediators such as eicosanoids, leukotrienes, and proteases, which further orchestrate the vascular leakage [8]. Dengue virus (DENV)-elicited tryptase promotes vascular permeability, and animal studies demonstrate that nafamostat mesylate, a tryptase inhibitor, can block DENV-induced vascular leakage, making it a possible therapeutic target [9]. Another emerging compound, NITD 688, is known to inhibit DENV protein NS4B in all 4 dengue serotypes and reduce viremia in preclinical animal models, thus hinting toward its potential role in dengue treatment [10]. Similarly, host-directed therapies such as α-glucosidase inhibitors reduce DENV replication in in vivo studies [11]. These studies emphasize the need for continued research to advance development of dengue diagnostic and therapeutic targets.

### Lessons from successful dengue control programs

In 2011, Lahore city of Pakistan experienced its most serious dengue epidemic with 496,490 suspected cases and 300 deaths overwhelming the healthcare infrastructure [12]. In response, a Central Emergency Response Committee integrating multidisciplinary efforts of health, agriculture, environment, and horticulture ministries was established [12]. A Dengue Expert Advisory Group identified suspected dengue patients referring them to public hospitals for standardized diagnostic test and treatment [13]. Data from each confirmed case was entered in a centralized patient tracking system [13]. Technological initiatives included implementation of online surveillance systems [12], global positioning systems [12], phone-based surveillance system [13], and toll-free help line for citizens [13]. Health infrastructure was revamped with establishment of isolation wards and high dependency units (HDUs), extra allocation of beds, and recruitment of healthcare professionals [12]. Isolation wards for dengue were established in 2011 not only in Lahore but also in other dengue-struck cities of the province of Punjab too and are in place to date. They were temporarily converted to COVID isolation wards in 2020 and 2021 in some healthcare facilities, where the load was very high of COVID patients. After the decline of COVID, their status has been reinstated. We also do have dedicated HDUs for dengue in tertiary care hospitals of major cities both public and private. Medical practitioners and public health managers received training from Sri Lanka and Thailand and acted as master trainers to train local professionals [12].

Semarang City in Indonesia, which reports one of the highest numbers of dengue cases in Central Java, is another example of successful dengue control program. To control the rising dengue incidence, the Governmental Health Office recruited dengue surveillance workers (DSWs) to improve environmental management through community participation, education, organization, and vector control. The success of DSWs work was demonstrated through the decline in dengue hemorrhagic fever (DHF) incidence from 92.4 per 100,000 inhabitants in 2014 to 18.14 per 100,000 inhabitants in 2017 [14], coexisting with 3 years in which DSWs were entrusted to work in the Semarang municipality [15]. “Tunggal Dara” is another unique dengue control program in Semarang City involving collaboration of Education Office, Ministry of Religion Affairs and Family Welfare, hospitals, nongovernment organizations, private sector, climatology and meteorology departments, and community.

Bangkok Children’s Hospital/Queen Sirikit National Institute of Child Health (QSNICH) serves as a standard for comparison of dengue case management and provides an excellent illustration of the role of clinical training in reducing case fatality rate (CFR). The QSNICH training programs on dengue case management have reduced CFR in several countries. For example, CFR reduced from 12% to 3.6% in Timor Leste, from 0.34% to 0.1% in Thailand, and from 0.7% to 0.3% in Cambodia [16]. More importantly, after the training, each country would need to develop their own guidelines for diagnosis, treatment, prevention, and control of dengue, which could be adopted from the ones of WHO or the other successful countries, and should get a consensus from multidiscipline experts.

Lessons from such successful dengue control programs should be considered as stepping-stones for developing dengue control strategies in other endemic settings. Collaborative multisectoral (health, education, environment, policy, and community) efforts are needed to build a strong dengue prevention framework to lead the global march against dengue. International expert online discussion and experience exchange by video conferences system to discuss and get the experts’ opinions about the treatments for complex and severe cases will also help reduce the CFR. ADVA group is attentive to this point then; they have also frequently held webinar series on dengue management to generate well-informed, focused, and coordinated actions across the Asia region, which may have a significant impact on dengue in Asia.

### Newer insights on varying dengue epidemiology

In a recent study, Sasmono and colleagues compared the clinical, demographic, and virological features of dengue in 3 cities—Batam, Banjarmasin, and Ambon representing Western, Central, and Eastern regions in Indonesia [17]. The median age of dengue confirmed patients was 15 years, 16 years, and 9 years in Batam, Banjarmasin, and Ambon, respectively. The percentage of patients with IgG antibodies on rapid diagnostic test (RDT) was higher in Banjarmasin (72.6%) compared to Ambon (55.8%) and Batam (22.0%). Approximately 84.9% of patients in Batam had dengue fever, while 60% patients in Ambon had DHF. The reported clinical symptoms and sensitivities of NS1, IgG, IgM RDTs and DENV serotype distribution also varied across the 3 cities. These results indicate that differences in geography, climate, demography, and quality of healthcare might contribute to the clinical, immunological, and virological differences in dengue epidemiology in Indonesia [17].

Another innovative study highlighting the variable dengue epidemiology is the ongoing, prospective, hybrid cohort cluster study in Kamphaeng Phet, Thailand, to illustrate DENV transmission in multigenerational households up to the year 2023 [18]. The study aims to compare the immunological profiles before and after infection with disease outcomes, define role of maternally derived DENV immunity in infants, define outcomes of sequential DENV infections in adults with prior exposures, and identify transmission risk factors in household units [18]. Such epidemiological studies are crucial for the development of unique dengue control strategies pertinent to local dengue epidemiology.

### Double challenge of dengue and COVID 19

The dual problem of Coronavirus Disease 2019 (COVID-19) caused by novel Severe Acute Respiratory Syndrome Coronavirus 2 (SARS-CoV-2) and DENV is an important public health issue, especially for developing countries that have overburdened public health infrastructure, economic obstacles, and limited diagnostic capability. Misdiagnosis and coinfection with both COVID-19 and dengue is a serious concern due to similar clinical and laboratory characteristics. Cases of coinfection have been reported in Singapore, Thailand, Malaysia, Brazil, and Southeast Asia [19]. Cross-reactivity between DENV and SARS-CoV-2 antibodies on serology tests, although unlikely, presents another diagnostic challenge. False positive dengue serological tests in COVID-19–positive patients were reported in Singapore [20] and Israel [21]. In Southeast Asia and South America, where both viruses are in cocirculation, 22% COVID-19 infections might be incorrectly diagnosed as dengue, complicating the management and treatment of both diseases [21]. A simple and accurate rapid test is essential to differentiate between COVID-19 and dengue [22]. In addition, a dramatic increase in the number of COVID cases caused almost all hospital beds to be occupied, limiting the accessibility to the healthcare system for those with non-COVID diseases, including dengue. The delay in diagnosis and treatment may also contribute to the increase in dengue fatality rates.

Another challenge encountered during the pandemic is the feasibility of continued vector control. After first 2 weeks of lockdown, Malaysia reported a sharp fall in dengue cases, followed by a rebound increase higher than the prepandemic level [23]. Similarly, Singapore [24], Peru, and Ecuador [25] registered increases in dengue cases during the lockdown. Interruption in vector control measures during the pandemic might have contributed to the rise in dengue cases [25]. It is essential to continue dengue vector surveillance as pre-COVID times and strengthen vector control interventions with an improved method [26]. Field-level health staff needs to be trained appropriately, follow physical distancing measures, and be provided with personal protective equipment (PPE) to confirm their safety [26]. Breeding sources should be monitored, and insecticides should be sprayed with the help of drones [26]. The public should be sensitized on dengue control and take preventive measures like reducing the breeding sources in their house and wearing protective clothing, using mosquito repellents, and using bed nets during daytime naps [26]. The pandemic highlights the pressing need for dengue preparedness through resource mobilization, environmental management, and the strengthening of public health infrastructure.

### Dengue vaccines

The challenge of dengue vaccine development is providing protective antibodies against all 4 dengue serotypes to avoid possibly causing an antibody-dependent enhancement in further infections [27]. Denvaxia is currently the only licensed vaccine with overall vaccine efficacy (60.3% to 60.8%) varying between DENV serotypes and age groups [28,29]. However, it raised serious safety concerns as it might increase the risk of hospitalization in dengue-naïve vaccinees when they are exposed to the virus [30]. At least 7 DENV vaccines have undergone different phases of clinical trials; however, Phase III clinical trials with only 2 vaccines, TV003/TV005 and TAK-003 (DENVAx), have shown promising results [27]. The cumulative efficacy data of the DENVax vaccine 3 years postvaccination shows an overall vaccine efficacy of 62% against virologically confirmed dengue. In baseline seronegative individuals, the DENVax effectiveness was 52.3% for DENV-3 and 83.4% for DENV-2. In baseline seronegative individuals, the vaccine was only effective against DENV-1 (43.5%) and DENV-2 (91.9%), but no efficacy was observed for DENV-3 [31]. The TV003/TV005 vaccine was licensed by the Butantan Institute in Brazil. The randomized placebo-controlled Phase II trial showed that it was safe and induced robust, balanced neutralizing antibody responses against the 4 DENV serotypes [32]. A Phase III clinical trial for the TV003/TV005 is currently being conducted in Brazil, and results for this trial are still not available [33]. A universal, highly effective, and safe dengue vaccine is imperative to improve its uptake [27].

Dengue vaccines, when they become available, should be integrated with mosquito control to prevent epidemic transmission. Although vaccinations are cost-effective public health measures, vaccine hesitancy is a global public health concern [34]. Factors contributing to vaccine hesitancy include complacency, inconvenience, safety, and lack of confidence [34]. Vaccine hesitancy has become a major issue more so during the COVID-19 pandemic due to spread of misinformation about the vaccines [35]. Continuous vaccine advocacy by engaging medical community [35], policy makers [35], public [36], and media [36] will be crucial in gaining public confidence and maintaining vaccination programs. Overcoming vaccine hesitancy will require coordinated communication strategies based on transparency timely credible science-based information and understanding of human behavior.

### Vector control

The control of *Aedes* mosquitoes is one of the options for the primary prevention of dengue [37]. Most vector control methods available for public health use are not very productive, not implemented to high quality, and not routinely implemented [38]. Therefore, dengue vector control aims to reduce vector densities as much as possible and maintain them at low levels [37]. Chemical methods widely used are organophosphates, such as Fenitrothion and Malathion, and different pyrethroid derivates. However, the use of chemical insecticides is declining because of the development of insecticide-resistant strains [39,40] and unforeseen side effects on human health and the ecosystem. Temephos [41], an organophosphorous substance, and Pyriproxyfen [42], a broad-spectrum insect growth regulator, are widely used for larviciding. Both chemicals are not toxic in drinking water when used with the prescribed dosage. However, they require a regular application. Novel vector control techniques such as the Incompatible Insect Technique (IIT) and the Sterile Insect Technique (SIT) are currently being studied for public health use [38]. IIT relies upon Wolbachia-infected male mosquitoes that cannot generate viable offspring after mating with a wild-type female [43,44]. Wolbachia can spread naturally in this population once released into the wild mosquito population. Similarly, SIT uses gamma-irradiation to produce large-scale random damage to the insect chromosomes [45–47]. When released into the wild, it can suppress and ultimately eliminate wild mosquito populations. However, this approach requires a constant release of irradiated sterile male insects that need substantial financial and human resources [48,49].

## Conclusions

To achieve the WHO 2021–2030 global target for dengue control, there is an urgent need for endemic countries to develop the infrastructure to support integrated, multisectoral prevention and control programs cosponsored by government at the local, state, and national levels (health, environment, and education ministries), the private sector, and the community. A World Dengue Day should be established to improve global awareness, share best practices and resources, and prioritize preparedness in the fight against dengue.
